# Maternal provisioning and zygotic activation: transcriptomic dynamics of early atlantic halibut (*Hippoglossus hippoglossus*) embryogenesis

**DOI:** 10.3389/fcell.2025.1723170

**Published:** 2025-12-08

**Authors:** Nils Niepagen, Francesca Bertolini, Leif Berg, Jonna Tomkiewicz, Elin Kjørsvik

**Affiliations:** 1 Department of Biology, Norwegian University of Science and Technology (NTNU), Trondheim, Norway; 2 Animal and Food Genomics Group, Division of Animal Sciences, Department of Agricultural and Food Sciences, University of Bologna, Bologna, Italy; 3 Nordic Halibut AS, Bergen, Norway; 4 National Institute of Aquatic Research, Technical University of Denmark, Henrik Dams Allé, Lyngby, Denmark

**Keywords:** atlantic halibut, embryonic development, transcriptomics, egg quality, maternal zygotic transition

## Abstract

**Introduction:**

In Atlantic halibut (*Hippoglossus hippoglossus*) aquaculture, egg and larval quality remain major bottlenecks. Most transcriptomic and proteomic studies compare freshly fertilized eggs of good versus poor quality, assuming early molecular differences explain hatching success. However, many lethal phenotypes arise only after the mid-blastula transition (MBT), suggesting that early comparisons may overlook key developmental processes.

**Methods:**

We performed stage-resolved RNA sequencing of Atlantic halibut embryos spanning unfertilized egg (UF), cleavage (8C, 16C), blastula (BL), 25% epiboly (25 EB), and blastopore closure (50 degree-days, 50 dd). Differential expression analysis (DESeq2 with LFC shrinkage), Gene Ontology (GO) overrepresentation (PANTHER, REVIGO), and curated axis-patterning gene sets from zebrafish orthologs were used to map transcriptional dynamics.

**Results:**

Minimal transcriptional change occurred during cleavage (UF–16C), dominated by maternal transcripts. A pronounced transcriptional shift emerged at MBT (16C→BL), marked by upregulation of canonical developmental pathways (Wnt, BMP, Notch) and axis-specification genes (e.g., *bmp2b, chrd, wnt8b, fgf8a*). These pathways remained active through gastrulation (BL→25 EB), alongside enrichment of morphogenetic processes such as mesoderm formation and left–right symmetry. By 50 dd, expression shifted toward lineage differentiation programs involving neurons, muscle, and cranial skeleton. Axis-regulator dynamics showed that dorso–ventral (D–V) patterning is initiated by maternal factors but reinforced by zygotic cascades, whereas anterior–posterior (A–P) patterning is primarily zygotic.

**Discussion:**

Our results demonstrate that the decisive transcriptional programs underlying axis formation and organogenesis are activated from MBT onward. These findings imply that comparisons limited to unfertilized or early cleavage stages cannot capture the biology determining embryo viability. Stage-resolved analyses encompassing MBT and gastrulation are therefore essential for identifying transcriptomic markers of egg and larval quality in halibut.

## 1 Introduction

Atlantic halibut (*Hippoglossus hippoglossus*) farming began in the 1990s in Norway, Scotland, and Iceland; today, Norway is the only country reporting substantial production (FAO, 2023). Yet major bottlenecks persist, especially inconsistent hatching success and high, variable rates of larval and juvenile deformities, which limit reliable supply of juveniles for grow-out (Brown, 2010; Engelsen et al., 2004; Lewis et al., 2004). These constraints echo observations across marine aquaculture species, where early developmental competence remains a key determinant of production efficiency. In Atlantic halibut specifically, Niepagen et al. (2025) showed that abnormalities observed before the mid-blastula transition (MBT) were only moderately predictive of hatching success, whereas defects emerging after MBT (e.g., absence of head structures, failed symmetry, impaired cell specification) were much stronger correlates of poor hatch outcomes.

At the molecular level, early vertebrate embryos undergo a major developmental shift during the MBT, when control of development passes from maternally provided transcripts and proteins to the newly activated zygotic genome. During this period, zygotic genome activation (ZGA) peaks as widespread transcription begins, cell cycles lengthen, and chromatin becomes more permissive to transcription. Understanding when specific developmental pathways are initiated is therefore crucial for interpreting phenotypes and designing informative sampling schemes (Lee et al., 2014; Vastenhouw et al., 2019).

In fish, stage-resolved transcriptomics has mapped these dynamics in several species, revealing modest transcriptional change during very early cleavages and extensive activation at or after MBT. Examples include zebrafish developmental series that confirmed limited pre-MBT transcription and sharp post-MBT activation, as well as carps and flatfishes where pathway-level reprogramming surrounds blastula/gastrulation (Duan et al., 2022; Fu et al., 2019; Vesterlund et al., 2011). Together, these studies underscore that many axis-setting and patterning genes are zygotically expressed.

Much of the aquaculture literature on finfish egg quality has focused primarily on differences assessed at fertilization or during early cleavage, and on transcript or protein abundance in unfertilized/freshly fertilized eggs (Mommens et al., 2010; Mommens et al., 2014; Yilmaz et al., 2022; Żarski et al., 2017). While these studies have been valuable, the Atlantic halibut case highlights a timing mismatch: Niepagen et al. (2025) found that post-MBT deformities, rather than pre-MBT variation, explained hatching success most effectively. If the decisive developmental programs only emerge around MBT, then transcriptomic contrasts among freshly fertilized eggs are poorly positioned to capture the biology most relevant for competence.

Here, we provide a stage-resolved transcriptomic atlas for early Atlantic halibut development, spanning unfertilized egg through cleavage to blastula and early gastrulation. The objectives of this study were to (i) map when major developmental pathways (e.g., Wnt/Notch/BMP, axis specification, morphogenesis) become over-represented, (ii) identify exemplar genes that define these windows, and (iii) supply a halibut-specific baseline that clarifies which comparisons are biologically meaningful. In doing so, we aim to place Atlantic halibut within the broader vertebrate framework of MZT/MBT timing and offer a rationale for stage-matched designs in future mechanistic and applied studies.

## 2 Materials and methods

### 2.1 Ethical statement

Fertilized eggs are developmental stages outside the scope of the Guidelines of the European Union on the protection of animals used for scientific purposes (Directive 2010/63/EU), and approval from the Norwegian Food Safety Authority was not needed. Broodstock handling was performed as part of common hatchery practice.

### 2.2 Sampling protocol

**FIGURE 1:**
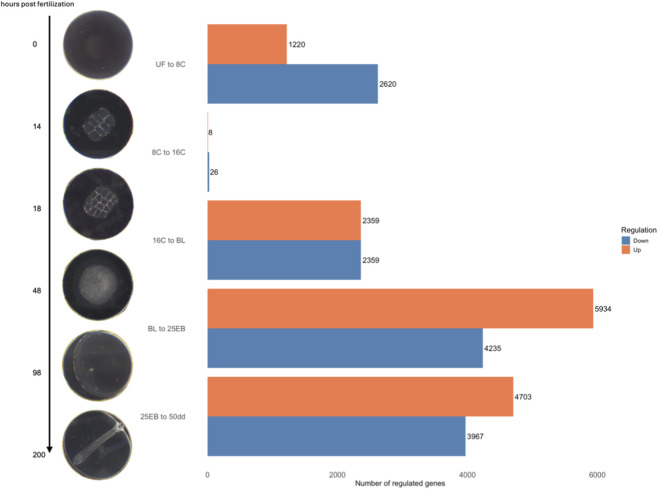
Overview of the Atlantic halibut (*Hippoglossus hippoglossus*) developmental stages and the number of differentially expressed genes (DEGs) across five developmental transitions spanning six stages. The representative images on the left illustrate these transitions chronologically, while the bar chart on the right displays the total number of significantly up- and downregulated genes for each transition. Blue bars indicate genes with higher expression changes in the less advanced developmental stage (downregulation), whereas orange bars represent genes with higher expression changes in the more advanced stage (upregulation). UF = unfertilized eggs, 8C = 8 cell stage, 16C = 16 cell stage, BL = blastula stage, 25 EB = 25% epiboly, 50 dd = 50-degree day stage. Developmental time at 6 °C.

Sampling occurred in the commercial hatchery at Nordic Halibut AS (Midsund, Norway), where good-quality egg batches from four females were collected at six developmental stages (Figure 1): unfertilized egg (UF), eight-cell (8C, 14 h post-fertilization, hpf), sixteen-cell (16C, 18 hpf), blastodisc (BL, 50 hpf), 25% epiboly (25% EB, 96 hpf), and blastopore closure (50 dd, 200 hpf). All developmental times are given in hours post-fertilization at 6 °C. For each developmental stage, three biological replicates were sampled, each derived from a distinct female. Female identities for all samples are provided in Supplementary Table S1. Egg batches were considered to be of good quality if they exhibited fertilization success >90% or hatching success >80%. Individual eggs were not assessed independently. The hatchery used a flow-through seawater system at 34 ppt salinity, cooled to 6 °C during the spawning season. Eggs were hand-stripped and immediately fertilized using cryopreserved milt (Cryogenetics AS, Norway), then incubated in commercial 250 L cylindro-conical incubators with light aeration and an upwelling flow of 6 °C seawater. For sampling, unfertilized eggs were collected immediately after being stripped from the females, and fertilized eggs were scooped out of the commercial incubators. All sampled eggs were transferred into 1.8 mL cryo-vials and immediately snap frozen in liquid nitrogen to preserve RNA integrity and stored at −80 °C until RNA extraction.

### 2.3 RNA extraction and sequencing

Total RNA was extracted from pools of ten eggs per sample using the RNeasy Universal Plus Mini Kit (Qiagen) according to the manufacturer’s protocol. RNA concentrations were quantified using NanoDrop spectrophotometer (NanoDrop Technologies, Santa Clara, CA). Samples were diluted to total RNA concentration of 200 ng/sample until estimation of RNA integrity numbers (RIN) for each sample that was calculated with the Bioanalyzer (Agilent Technologies, Santa Clara, CA). The average RIN value was 9.8 ± 0.24. Paired-end 150bp mRNA sequencing was then performed by the same company using Illumina HiSeq 2,500 platform (Illumina Inc., USA) following the manufacturer’s instruction at Novogene co (Beijing, China).

### 2.4 Data generation, data curation and differential expression

Read quality was assessed using FastQC (Andrews, 2010). Raw reads were inspected with FastQC on a representative subset of libraries, which consistently showed depressed per-base quality and/or primer/adapter carryover in the first 10 nucleotides (nt) at the 5′end. To mitigate this systematic artifact, we applied a fixed 10-nt 5′hard trim to all reads prior to alignment (no additional quality/adaptor trimming was performed) using Trimmomatic v0.38 (Bolger et al., 2014). TopHat2 v2.0.13 (Kim et al., 2013) was used to map the trimmed reads to the most recent Atlantic halibut reference genome and corresponding known transcripts (NCBI, fHipHip1. pri; GCF_009819705.1). Uniquely mapped reads were retained and sorted by name using Samtools v1.10 (Li et al., 2009). HTSeq-count (Anders et al., 2015) was used to generate read counts for each annotated gene in every sample, using the intersection-strict mode to exclude ambiguously mapped reads and assuming unstranded RNA-seq data. All 18 samples (three biological replicates per developmental stage) yielded complete count data with no missing values, and all libraries passed quality control and were included in the final DESeq2 analysis. The differential gene expression was performed using the DESeq2 package for R (Love et al., 2014) using all sample counts for data normalization. Differential expression analysis was performed by comparing each developmental stage with its subsequent stage (UF vs. 8C, 8C vs. 16C, 16C vs. BL, BL vs. 25 EB, and 25 EB vs. 50 dd). P-values were adjusted for multiple testing using the Benjamini–Hochberg method to control the false discovery rate (FDR), and genes with FDR < 0.05 were considered significantly differentially expressed and included in the gene overrepresentation analysis (Benjamini and Hochberg, 1995). Each transition was divided into up- and downregulated genes based on the sign of the shrunken log_2_ fold change (LFC), where positive values indicate higher expression in the more developed stage and negative values indicate higher expression in the less developed stage. Statistical significance was assessed with DESeq2 Wald tests and p-values were adjusted for multiple testing using the Benjamini–Hochberg procedure (FDR); genes with FDR (padj) < 0.05 were considered differentially expressed. Log_2_ fold changes were then shrunk after model fitting using lfcShrink with the ashr method to reduce the influence of technical noise and improve effect-size stability (Stephens, 2017).

### 2.5 Statistical overrepresentation analysis (ORA) and relevant GO terms

For each pairwise comparison, overrepresentation analysis (ORA) was performed with the differentially expressed genes, divided in upregulated and downregulated using Panther (http://www.pantherdb.org/) and *Danio rerio* as reference species. Here, GO term biological processes were considered, with False discovery rate FDR (padj) < 0.05. Redundant GO terms were eliminated using REVIGO (Supek et al., 2011) with the *D. rerio* database. Redundant GO terms are defined as terms that convey similar biological meaning to another term in the result list, usually because they share many of the same genes and exist in a hierarchical structure. The removal process involved SimRel as the semantic similarity measure with a cut-off value of 0.5. To refine the overrepresentation analysis, a cut-off was applied to exclude overly general GO terms that were present in more than 10% of the annotated genes.

Based on the overrepresentation results, the following curated subset of Gene Ontology (GO) terms was highlighted as regulators of embryonic development: GO:0050793 (regulation of developmental process), GO:0048598 (embryonic morphogenesis), GO:0030510 (regulation of BMP signaling pathway), GO:0016055 (Wnt signaling pathway), GO:0009790 (embryo development), GO:0007498 (mesoderm development), GO:0007389 (pattern specification process), GO:0007368 (determination of left/right symmetry), GO:0007219 (Notch signaling pathway), and GO:0007186 (G protein-coupled receptor signaling pathway). These terms reflect core developmental pathways broadly implicated in vertebrate embryogenesis (Gilbert and Michael, 2019) and were selected for their repeated overrepresentation across developmental transitions and their established roles in embryonic patterning and axis specification during early vertebrate development. To visualize the temporal dynamics of transcriptional activation of these processes, –log10 (p-value) scores from overrepresentation analysis were plotted across developmental transitions (UF to 8C, 8C–16C, 16C to BL, BL to 25 EB, and 25 EB to 50 dd), reflecting the statistical significance of GO term overrepresentation for the selected GO terms at each transition. This approach provided insight into the developmental timing of significant overrepresentation for each selected pathway, serving as a proxy for the onset of transcriptional activity.

A curated list of 109 developmental genes with known functions in vertebrate embryogenesis was compiled from zebrafish (*D. rerio*) developmental reviews (Fuentes et al., 2020; Schier and Talbot, 2005). Genes were grouped into two categories: dorso–ventral (D–V) patterning and anterior–posterior (A–P) patterning (Table 1). Atlantic halibut orthologs were identified by manual searches of the NCBI database (BLAST and gene annotations). Gene expression dynamics across developmental stages were visualized as heatmaps using log10-transformed, variance-stabilized counts from DESeq2, standardized to z-scores across samples and plotted with the ComplexHeatmap R package. (Gu, 2022).

**TABLE 1:** Atlantic halibut (*Hippoglossus hippoglossus*) genes with orthologs involved in zebrafish embryonic development. D–V patterning includes genes with known functions in dorso–ventral patterning, and A–P patterning includes genes with known functions in anterior–posterior patterning and left–right symmetry. The gene listed corresponds to the zebrafish ortholog identified in the NCBI Gene database. Function provides a brief description of each gene’s mode of action, and Phenotype summarizes the zebrafish (*Danio rerio*) knockout mutant phenotype. References indicate key studies describing each gene’s developmental role in zebrafish. Genes and references are based on two reviews of zebrafish development (Fuentes et al., 2020; Schier and Talbot, 2005).

Gene name	Function	Phenotype	References
D-V patterning			
*bmp2b*	Bmp signal	Severely dorsalized	Kishimoto et al. (1997), Schmid et al. (2000)
*bmp7b*	Bmp signal	Severely dorsalized	Dick et al. (2000), Schmid et al. (2000)
*acvr1l*	Type I Bmp receptor	Severely dorsalized	Bauer et al. (2001), Mintzer et al. (2001), Mullins et al. (1996)
*smad5*	Transcription factor	Weakly (zyg.) or strongly (mat.) dorsalized	Hild et al. (1999)
*twsg1a*	Bmp agonist	Dorsalized	Little and Mullins (2004), Xiao et al. (2005)
*neto1l*	Metalloprotease for Chordin	Weakly dorsalized	Connors et al. (1999)
*chrd*	Bmp inhibitor	Ventralized	Langdon and Mullins (2011), Schulte-Merker et al. (1997), Zinski et al. (2018)
*szl*	Bmp inhibitor	Ventralized	Martyn and Schulte-Merker (2003), Yabe et al. (2003)
*gdf6a*	Bmp signal	Dorsalized	Sidi et al. (2003)
*admp*	Divergent Bmp signal	Dorsalized	Lele et al. (2001), Willot et al. (2002)
*sp5a*	SP1 Zn Finger	Anteriorized and dorsalized	Weidinger et al. (2005)
*sp5l*	SP1 Zn Finger	Anteriorized and dorsalized	Weidinger et al. (2005)
*ctnnb1*	B-catenin localization	Variably ventralized	Kelly et al. (2000), Valenti et al. (2015)
*sybu*	B-catenin stability?	Variably ventralized	Mei et al. (2009), Nojima et al. (2010), Nojima et al. (2004)
*wnt8b*	Wnt signal	No ventral and posterior structures	Erter et al. (2001), Hino et al. (2018), Lekven et al. (2001)
*fgf8a*	FGF signal	Ventralized with loss of chordin	Fürthauer et al. (2004), Reifers et al. (1998)
*il17rd*	Antagonist of FGF signaling	Dorsalized	Fürthauer et al. (2002), Tsang et al. (2002)
*spry2*	Antagonist of FGF signaling	Dorsalized	Fürthauer et al. (2004)
*dusp6*	Antagonist of FGF signaling	Dorsalized	Tsang et al. (2004)
*vox*	Transcriptional repressor	Severely doralized in double mutants	Imai et al. (2001), Ramel and Lekven (2004), Shimizu et al. (2002)
*vent*	Transcriptional repressor	Severely doralized in double mutants	Imai et al. (2001), Ramel and Lekven (2004), Shimizu et al. (2002)
*ved*	Transcriptional repressor	Severely dorsalized with vox/vent	Imai et al. (2001), Ramel and Lekven (2004), Shimizu et al. (2002)
*prdm1a*	Transcriptional repressor	Dorsalized	Wilm and Solnica-Krezel (2005)
*ptbp1a*	Intracellular Ca2+ regulation	Dorsoventral axis formation defect	Mei et al. (2009)
*hwa*	transmembrane protein, mediated by Syntabulin	no dorsal organizer, strongly ventralized, huluwa can induce a dorsal axis	Shinya et al. (2000), Yan et al. (2018)
*cav1*	β-catenin regulation	Ventralized	Mo et al. (2010)
*wnt6b*	Dorsal determinant candidate - similar protein architecture	overexpression: dorsalization	Hino et al. (2018)
*tob1a*	β-catenin regulation	inhibits dorsal cell fate induction	Mo et al. (2010), Xiong et al. (2006)
*ccr7*	β-catenin regulation	Ventralized	Wu et al. (2012)
*rspo3*	Wnt signal	inhibits ventralizing zygotic Wnt signaling	Rong et al. (2014)
*ints6*	Bmp signal	strongly dorsalized, with secondary dorsal axes	Kapp et al. (2013)
*foxo3b*	bind to maternal β-catenin	dorsalized	Li et al. (2011), Liu et al. (2018), Xie et al. (2011)
*eaf1*	bind to maternal β-catenin	dorsalized	Li et al. (2011), Liu et al. (2018), Xie et al. (2011)
*eaf2*	bind to maternal β-catenin	dorsalized	Li et al. (2011), Liu et al. (2018), Xie et al. (2011)
*ctsba*	endopeptidase, regulates DV patterning	dorsalized	Langdon et al. (2016)
*spry4*	FGF antagonists	weak dorsalization	Fürthauer et al. (2001)
*fsta*	BMP antagonists	Ventralized if kock out together with noggin	Langdon and Mullins (2011), Zinski et al. (2018)
*gsc*	BMP antagonists	Ventralized	Joore et al. (1996), Kawahara et al. (2000), Kuo et al. (2013), Langdon and Mullins (2011), Maegawa et al. (2006), Zinski et al. (2018)
*zbtb4*	transcription factor, maternally- deposeted, initiates wnt8a transcription	strongly dorsalized	Yao et al. (2010)
*fgfr2*	FGF signal	anteriorized and dorsalized embryo	Challa and Chatti (2013), Rohner et al. (2009), White et al. (2017)
*dazl*	Germline transcript	DV defects	Welch and Pelegri (2015)
*bmpr1ba*	Bmp signal	interrupted DV patterning	Little and Mullins (2009), Smith et al. (2011)
*bmpr1bb*	Bmp signal	interrupted DV patterning	Little and Mullins (2009), Smith et al. (2011)
*bmp1a*	BMP antagonist	non-DV patterning embryo	Connors et al. (1999), Van Eeden et al. (1996)
A-P and R-L Patterning			
*wnt8b*	Wnt signal	No ventral and posterior structures	Erter et al. (2001), Hino et al. (2018), Lekven et al. (2001)
*sp5a*	SP1 Zn Finger	Anteriorized and dorsalized	Weidinger et al. (2005)
*sp5l*	SP1 Zn Finger	Anteriorized and dorsalized	Weidinger et al. (2005)
*fgf24*	FGF signal	Loss of posterior structures with loss of fgf8	Draper et al. (2003)
*gsk3ba*	required for vegetal pole microtubule organization	AP patterning	Schneider et al. (2012)
*dvl2*	required in AP-patterning	anteriorized, cyclopic embryos	Xing et al. (2018)
*fgfr1a*	FGF signal	anteriorized	Challa and Chatti (2013), Rohner et al. (2009), White et al. (2017)
*fgfr1b*	FGF signal	anteriorized	Challa and Chatti (2013), Rohner et al. (2009), White et al. (2017)
*fgfr2*	FGF signal	anteriorized and dorsalized embryo	Challa and Chatti (2013), Rohner et al. (2009), White et al. (2017)
*fgfr3*	FGF signal	anteriorized	Challa and Chatti (2013), Rohner et al. (2009), White et al. (2017)
*fgfr4*	FGF signal	anteriorized	Challa and Chatti (2013), Rohner et al. (2009), White et al. (2017)
*smad4a*	Transcription factor	Interrupted AP patterning	Hata and Chen, 2016; Hill (2016)
*dand5*	Antagonist of Nodal signaling	Loss of LR asymmetry	Hashimoto et al. (2004)
*spaw*	Nodal signal	Loss or randomization of LR asymmetry	Long et al. (2003)

Atlantic halibut orthologs of known zebrafish zygotic genome activators (*nanog*, *pou5f3*, *sox19b*, *sox2*, *sox3*) were identified and their rlog-normalized expression values extracted across all developmental stages. Mean expression and standard error were calculated per stage (n = 3 biological replicates). Maternal versus zygotic expression classification. The 58 unique developmental patterning genes from Table 1 were classified based on their expression dynamics. Genes were considered maternally provided if rlog-normalized expression in unfertilized eggs exceeded six and did not increase >2 units at later stages. Genes were classified as zygotically activated if expression increased >2 units from unfertilized to any post-MBT stage (BL, 25 EB, or 50dd). Genes meeting both criteria were classified as maternal + zygotic. Peak expression stage was determined as the developmental stage with maximum rlog-normalized expression.

## 3 Results

**FIGURE 2:**
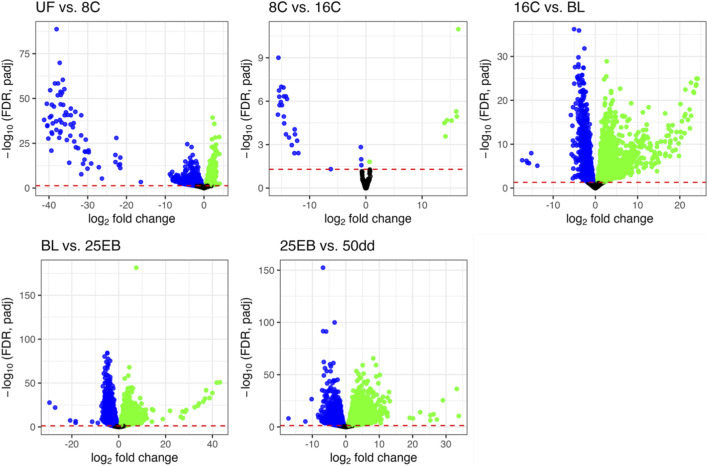
Distribution of differentially expressed genes. Each plot illustrates the transition from one sampled developmental stage to the next. Non-significant genes (padj ≥0.05) are shown in black; genes with higher expression in the more advanced stage are shown in green; and genes with higher expression in the earlier stage are shown in blue. The y-axis shows–log_10_(FDR, padj), and the x-axis shows log_2_ fold change between stages. UF = unfertilized eggs, 8C = 8 cell stage, 16C = 16 cell stage, BL = blastula stage, 25 EB = 25% epiboly, 50 dd = 50-degree day stage.

After producing and trimming the RNA-seq data, 25374672 ± 2216047 high quality reads per sample remained and were used for mapping. Approximately 88.8% ± 0.8% reads were successfully aligned against the Atlantic halibut reference genome and annotated genes (see Supplementary Table S1 for sample-specific alignment success). An overview of the developmental stages and the corresponding number of differentially expressed genes (DEGs) between transitions is shown in Figure 1. The distributions of the DEGs, including their -log10 transformed p-values and their log_2_ fold change is shown in Figure 2.

A total of 28793 significantly differentially expressed genes (FDR (padj) < 0.05) were retained across all pairwise stage comparisons (Figure 1). This number represents the sum of significant genes across transitions, and individual genes may appear in more than one comparison (Supplementary Table S2). The distribution of differentially expressed genes was as follows: 3,840 in the first transition (UF vs. 8C), 34 in the second (8C vs. 16C), 6,080 in the third (16C vs. BL), 10169 in the fourth (BL vs. 25 EB), and 8,670 in the final transition (25 EB vs. 50 dd). Hereafter, upregulation refers to genes with higher expression in the more advanced developmental stage, and downregulation to genes with higher expression at the earlier stage. Among these, 1,220 genes were upregulated (31.8%) and 2,620 downregulated (68.2%) in the first transition (UF vs. 8C). In the second transition (8C vs. 16C), only 8 genes (23.5%) were upregulated, while 26 (76.5%) were downregulated. The third transition (16C vs. BL) showed a balanced distribution of 2,359 upregulated (50%) and 2,359 downregulated (50%) genes. In the fourth transition (BL vs. 25 EB), 5,934 genes were upregulated (58.3%) and 4,235 downregulated (41.7%). In the final transition (25 EB vs. 50 dd), 4,703 genes were upregulated (54.2%) and 3,967 downregulated (45.8%). This distribution indicates a generally balanced pattern, with some transitions skewed toward greater up- or downregulation. Minimal gene expression changes were observed between the earliest cleavage stages, which are governed primarily by maternally derived transcripts. A marked increase in differential expression begins at the blastula stage, coinciding with the onset of the mid-blastula transition (MBT).

**FIGURE 3:**
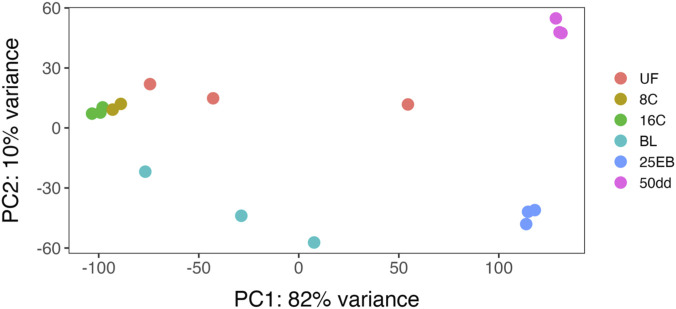
Principal component analysis (PCA) of the samples from several Atlantic halibut (*Hippoglossus hippoglossus*) developmental stages based on log2 transformed transcriptomic values by developmental stage. UF = unfertilized eggs, 8C = 8 cell stage, 16C = 16 cell stage, BL = blastula stage, 25 EB = 25% epiboly, 50 dd = 50-degree day stage.

Principal component analysis (PCA) of the variance-stabilized gene expression data across developmental stages, as computed in DESeq2, is shown in Figure 3. Unfertilized eggs and the blastula stage did not show clear groupings, whereas 8 and 16 cell stage formed two clusters, both close within and towards each other. Both the 25% epiboly stage and the subsequent 50 dd stage formed distinct and distant clusters. Hence, despite some variability within the groups, the developmental stage of the samples played a pivotal role in the clustering and variance of the transcriptomic profile.

### 3.1 Gene set enrichment analysis

**FIGURE 4:**
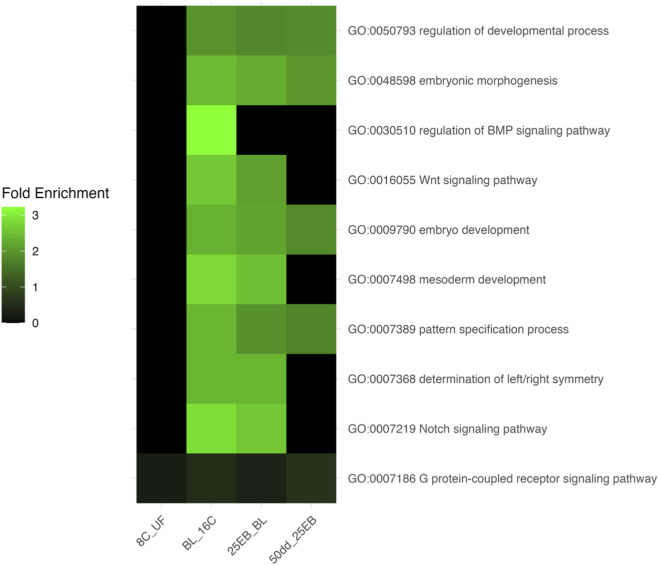
Heatmap of fold enrichment values for selected developmental Gene Ontology (GO) terms across transitions in Atlantic halibut (*Hippoglossus hippoglossus*) embryonic development. Each row represents a GO term, labeled as “Term ID – Name,” and each column corresponds to a developmental transition. The color gradient indicates the degree of GO-term enrichment (blue = low, green = moderate, black = baseline). UF = unfertilized eggs, 8C = eight-cell stage, 16C = sixteen-cell stage, BL = blastula stage, 25 EB = 25% epiboly, 50 dd = 50-degree-day stage.

The fold enrichment values for selected developmental Gene Ontology (GO) terms across the five developmental transitions are shown in Figure 4. No biological processes were enriched in the transition from 8C to 16C, reflecting the overall similarity in gene expression profiles during the early cleavage stages. In contrast, a pronounced increase in the enrichment of key developmental processes was observed from the blastula stage onward, particularly in the transition from 16C to BL and continuing through to 25 EB. During this window, pathways such as Wnt, BMP, Notch signaling, and pattern specification became significantly enriched, consistent with activation of the zygotic genome and the onset of embryonic patterning. In the last transition (25 EB to 50 dd), only more general developmental terms remained significantly enriched, indicating that the peak transcriptional engagement of canonical developmental pathways likely occurs during or immediately following the mid-blastula transition (MBT). This enrichment pattern suggests that, in the present analysis, maternal transcripts contributed only minimally to the expression of these selected developmental pathways. This interpretation is supported by the absence of enriched developmental GO terms during the early cleavage stages, when maternal mRNAs dominate, and the strong enrichment of such terms beginning at the blastula stage, coinciding with zygotic genome activation.

### 3.2 GO term overrepresentation in developmental transitions

**FIGURE 5:**
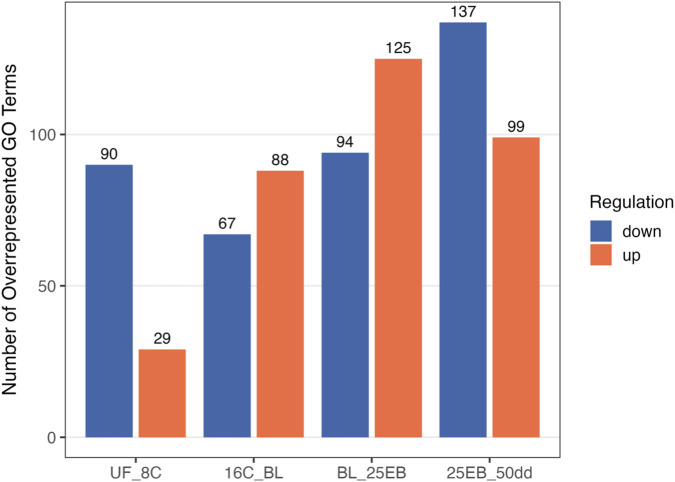
Number of significantly overrepresented Gene Ontology (GO) biological processes across developmental transitions in Atlantic halibut (*Hippoglossus hippoglossus*). Bars represent GO terms enriched among upregulated (coral) and downregulated (steelblue) genes in each pairwise stage comparison. Upregulated refers to GO terms that are expressed in the more advanced developmental transition, whereas downregulated refers to GO terms with higher expression in the earlier embryonic stage. Transitions are ordered developmentally from unfertilized egg (UF) to 50-degree days (50dd). UF = unfertilized eggs, 8C = 8 cell stage, 16C = 16 cell stage, BL = blastula stage, 25 EB = 25% epiboly, 50 dd = 50-degree day stage.

The overrepresentation analysis identified a total of 2,911 overrepresented Gene Ontology (GO) biological terms across the various developmental transitions (see Supplementary Table S3 for GO terms and associated genes). ORA tests whether certain biological functions are represented more frequently in a list of differentially expressed genes than would be expected by chance, helping to reveal underlying biological themes. GO terms were filtered using REVIGO to reduce redundancy and improve interpretability, and all results reported here refer to this redundancy-reduced set. After filtering, 729 non-redundant terms were retained for further analysis: 341 were associated with upregulated genes (i.e., higher expression in the later developmental stage), and 388 with downregulated genes (i.e., higher expression in the earlier developmental stage) (Supplementary Table S4; Figure 5). While both up- and downregulated GO terms were identified through overrepresentation analysis, only upregulated terms, those that are overrepresented in the more developed embryonic stages, were further analyzed in detail, as the focus was on the activation of developmental pathways during embryogenesis. The number of downregulated terms per transition is reported for completeness.

#### 3.2.1 Unfertilized eggs to eight and 16 cell stages

**FIGURE 6:**
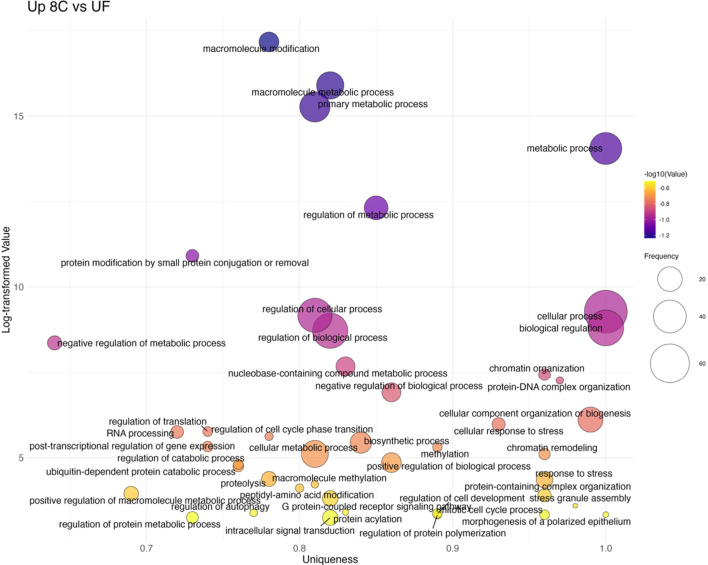
Bubble plot of non-redundant GO terms with higher expression in 8C stage eggs compared to UF eggs of Atlantic halibut (*Hippoglossus hippoglossus*). Frequency refers to the number of genes with signficant changes in expression, associated with each GO term, uniqueness assesses how distinct a term is in relation to the entire list, based on semantic comparison. Terms with higher uniqueness values are typically less likely to be redundant or dispensable. Log-transformed values are reversed (-log10 transformed) p-values, where larger values indicate higher significance. UF = unfertilized eggs, 8C = 8 cell stage.

The first transition (UF→8C) revealed 29 upregulated GO terms (Figure 6; Supplementary Table S4). These were dominated by stress- and signaling-related categories such as response to stress (GO:0006950), G protein–coupled receptor signaling pathway (GO:0007186), and chromatin organization (GO:0006325). In contrast, 90 terms had higher expression in unfertilized eggs, including several developmental processes such as embryo development (GO:0009790), mesoderm development (GO:0007498), and regulation of developmental process (GO:0050793). No significant overrepresentation was observed in the subsequent 8C→16C transition, consistent with the overall stability of the cleavage stages.

#### 3.2.2 16 cell stage to blastula stage

**FIGURE 7:**
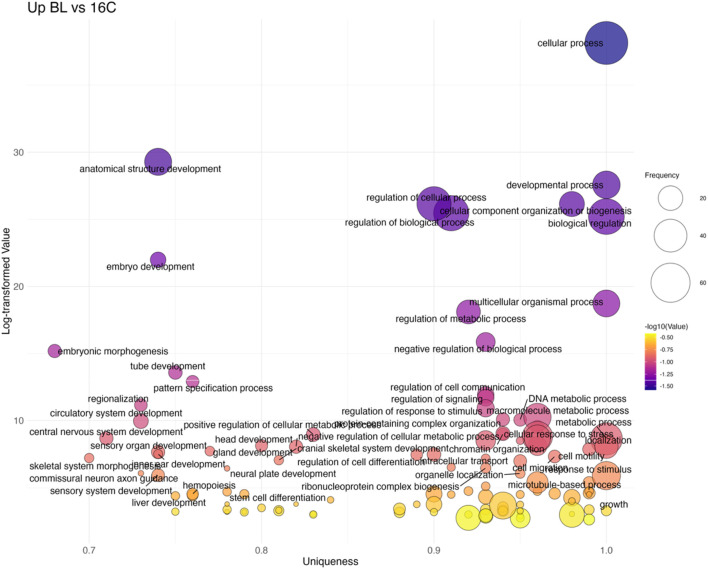
Bubble plot of non-redundant GO terms with higher expression in BL stage eggs compared to 16C eggs of Atlantic halibut (*Hippoglossus hippoglossus*). Frequency refers to the number of genes with signficant changes in expression, associated with each GO term, uniqueness assesses how distinct a term is in relation to the entire list, based on semantic comparison. Terms with higher uniqueness values are typically less likely to be redundant or dispensable. Log-transformed values are reversed p-values, where larger values indicate higher significance. 16C = 16 cell stage, BL = blastula stage.

The transition from 16C to BL marked the first strong transcriptional shift, with 88 non-redundant GO terms upregulated (Figure 7; Supplementary Table S4). Key processes included embryo development (GO:0009790), embryonic morphogenesis (GO:0048598), and axis-patterning pathways such as Wnt signaling (GO:0016055) and Notch signaling (GO:0007219), consistent with the onset of zygotic transcription at MBT. Several stress- and signaling-related terms were also overrepresented, reflecting broad cellular reprogramming. In contrast, processes related to cell cycle (GO:0007049) and cellular localization (GO:0051641) were downregulated, indicating a shift from cleavage divisions toward morphogenetic patterning.

#### 3.2.3 Blastula stage to 25% epiboly

**FIGURE 8:**
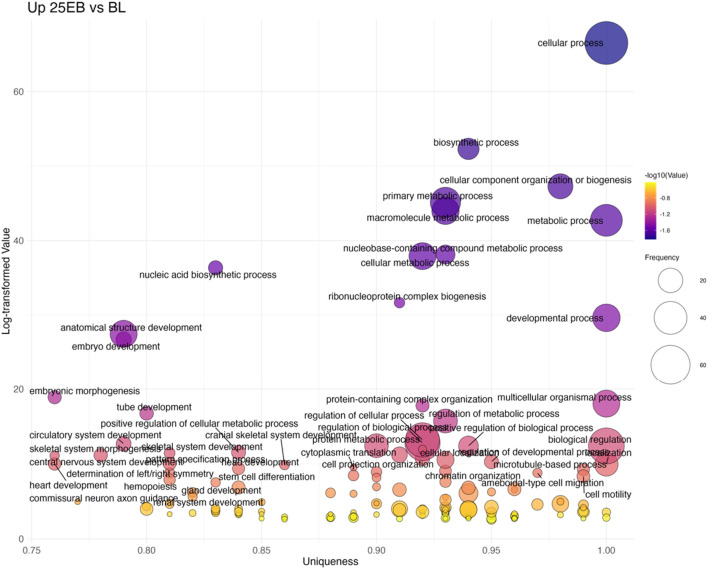
Bubble plot of non-redundant GO terms with higher expression in 25 EB stage eggs compared to BL stage eggs of Atlantic halibut (*Hippoglossus hippoglossus*). Frequency refers to the number of genes with signficant changes in expression, associated with each GO term, uniqueness assesses how distinct a term is in relation to the entire list, based on semantic comparison. Terms with higher uniqueness values are typically less likely to be redundant or dispensable. Log-transformed values are reversed p-values, where larger values indicate higher significance. BL = blastula stage, 25 EB = 25% epiboly.

The BL→25 EB transition showed the largest number of upregulated terms (125 total; Figure 8; Supplementary Table S4). Overrepresented categories included broad developmental processes such as embryo development (GO:0009790), embryonic morphogenesis (GO:0048598), and pattern specification (GO:0007389). More specialized terms were also prominent, including determination of left/right symmetry (GO:0007368) and mesoderm development (GO:0007498), alongside continued overrepresentation of Wnt signaling (GO:0016055). These results indicate that gastrulation is accompanied by both general morphogenetic activity and the specification of body axes.

#### 3.2.4 25% epiboly to 50 dd stage

**FIGURE 9:**
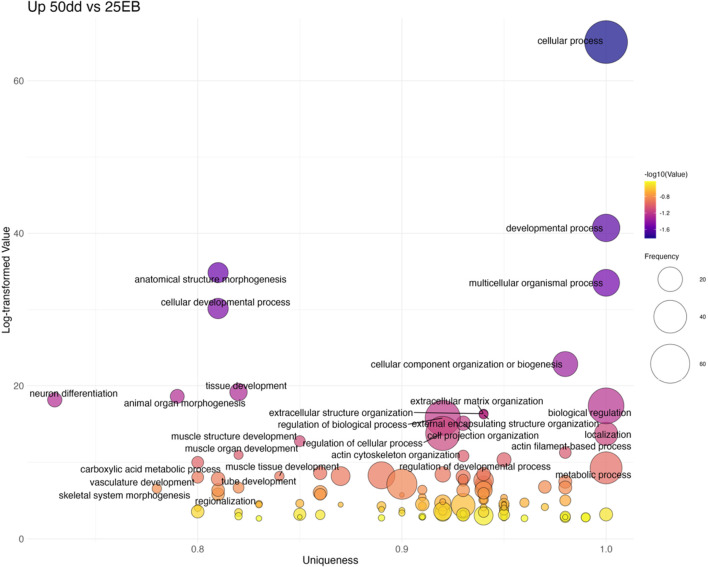
Bubble plot of non-redundant GO terms with higher expression in 50dd stage eggs compared to 25 EB stage eggs of Atlantic halibut (*Hippoglossus hippoglossus*). Frequency refers to the number of genes with signficant changes in expression, associated with each GO term, uniqueness assesses how distinct a term is in relation to the entire list, based on semantic comparison. Terms with higher uniqueness values are typically less likely to be redundant or dispensable. Log-transformed values are reversed p-values, where larger values indicate higher significance. 25EB = 25% epiboly, 50 dd = 50° day stage.

The final transition (25 EB→50 dd) was characterized by 99 upregulated GO terms (Figure 9; Supplementary Table S4). These included more general developmental categories such as tissue development (GO:0009888), animal organ morphogenesis (GO:0009887), and pattern specification (GO:0007389). Several lineage-specific processes also emerged, including neuron differentiation (GO:0030182), muscle structure development (GO:0061061), and cranial skeletal system development (GO:1904888). Some categories, including head development (GO:0060322) and regionalization (GO:0003002), appeared as both up- and downregulated terms, reflecting the dynamic refinement of transcriptional programs at this stage. Overall, these data suggest that while early patterning pathways peak at MBT and gastrulation, later stages shift toward differentiation and organogenesis.

### 3.3 Overrepresentation of key developmental GO terms

No developmental GO terms were significantly overrepresented before MBT (UF→8C, 8C→16C). From the 16C→BL transition onward, broad developmental categories such as embryonic morphogenesis, embryo development, and pattern specification process became significantly overrepresented and remained overrepresented through gastrulation. Canonical signaling pathways including Wnt, Notch, and BMP were specifically overrepresented in the 16C→BL and BL→25 EB transitions, coinciding with the onset of zygotic transcription. By 50 dd, developmental GO terms were no longer significantly overrepresented, consistent with a shift toward later differentiation programs. Of the curated developmental genes analyzed in Section 3.3, 18 mapped directly to these GO terms (Table 2), linking pathway-level changes to individual gene dynamics.

**TABLE 2:** Developmental Gene Ontology (GO) terms and their associated Atlantic halibut (*Hippoglossus hippoglossus*) gene sets used for gene set enrichment analysis (GSEA). Each term represents a curated developmental process, with associated genes extracted from the Atlantic halibut (*Hippoglossus hippoglossus*) dataset based on zebrafish orthologs.

GO term	Associated genes
GO:0007368 - determination of left/right symmetry	*bmp2b, chrd, fgf8a, fgfr2, foxh1, spaw, tbxta, wnt11*
GO:0007498 - mesoderm development	*bmp2b, dact2, fgf8a, fgfr1a, fgfr1b, fgfr2, foxh1, spaw, tbxta*
GO:0016055 - Wnt signaling pathway	*ctnnb1, dact2, sfrp2l, tbxta, tcf3a, tcf3b, vangl2, wnt11, wnt8b*
GO:0030510 - regulation of BMP signaling pathway	*bmp2b, chrd, smad4a, tbxta*

### 3.4 Genes with known developmental function in vertebrate embryonic development

**FIGURE 10:**
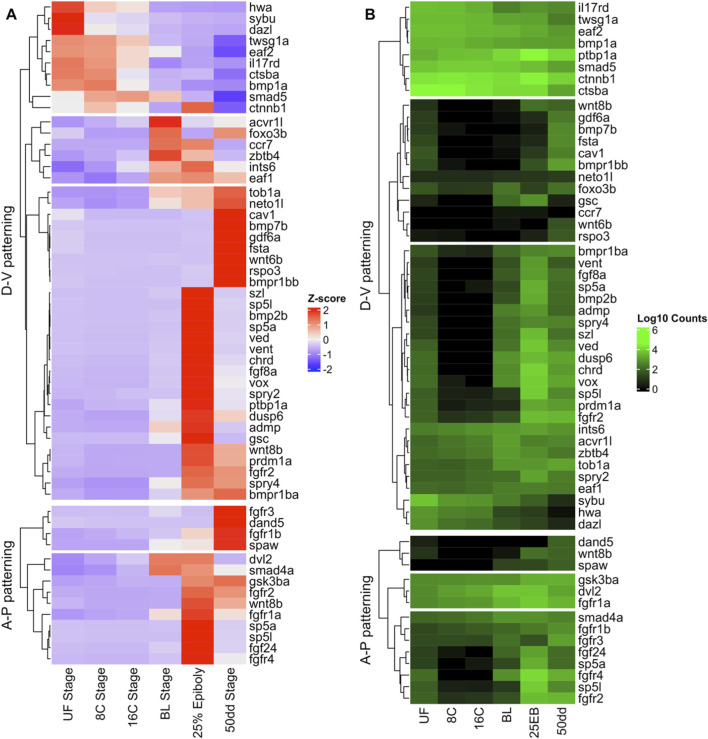
Differential expression of developmental genes during Atlantic halibut (*Hippoglossus hippoglossus*) embryonic development. **(A)** Z-score–transformed normalized gene counts for each sample. A positive Z-score indicates high expression relative to stages with lower Z-scores, illustrating transcriptional differences across developmental stages. **(B)** Log_10_-transformed normalized counts demonstrating each gene’s expression magnitude relative to other genes. D–V patterning genes function in the establishment of the dorso–ventral axis, while A–P patterning genes control determination of the anterior–posterior axis and left/right asymmetry. UF = unfertilized eggs; 8C = eight-cell stage; 16C = sixteen-cell stage; BL = blastula stage; 25 EB = 25% epiboly; 50 dd = 50-degree-day stage (blastopore closure).

In contrast to the genome-wide GO overrepresentation analysis in Section 3.1, we next focused on stage-wise expression trajectories for two canonical developmental gene sets, dorso–ventral (D–V) and anterior–posterior (A–P) axis regulators, curated from the literature (Table 1; Figure 10). Across these genes we observed three recurrent temporal profiles: (i) UF→16C biased (“maternal”) expression followed by decline after MBT, (ii) activation at BL and/or 25% EB (MBT/early gastrulation), and (iii) biphasic patterns with BL activation, a dip at 25% EB, and reactivation toward 50 dd. These trajectories mirror the GO results (Section 3.1), where early patterning terms rise at BL/25% EB and differentiation terms dominate by 50 dd.

#### 3.4.1 Dorso-ventral patterning

Many D–V regulators (e.g., *ctnnb1, hwa, sybu*) are highly expressed in unfertilized eggs and cleavage-stage embryos and decline after MBT, consistent with maternal provisioning (Figure 10A). A larger group (e.g., *bmp2b, sp5a, chrd, fgf8a*) is low pre-MBT but switches on at BL/25% EB, reflecting zygotic activation of morphogenetic signaling. A smaller subset (e.g., *wnt8b*, *prdm1a*) shows biphasic behavior, activation at BL, attenuation at 25% EB, and renewed expression at 50 dd, suggesting later roles during differentiation. Overall, D–V specification appears to be initiated by maternal factors and reinforced by zygotic BMP/FGF/Wnt cascades after MBT (Figures 10A,B).

#### 3.4.2 Anterior-posterior patterning

In contrast, most A–P regulators were transcriptionally silent before MBT and were activated only at BL (e.g., *sp5a, sp5l, wnt8b, fgf24*). Some, such as *dvl2* and *smad4a*, peaked at BL and persisted into 25 EB, while others (e.g., *fgfr3, spaw*) were activated only later at 50 dd. This indicates that A–P axis establishment in halibut is primarily a zygotic process, initiated during MBT and refined through gastrulation into later organogenesis (Figures 10A,B).

### 3.5 Maternal-zygotic dynamics of key developmental regulators

Atlantic halibut orthologs of the zebrafish zygotic genome activators *nanog*, *pou5f3* (*pou5f1*), and the SoxB1 genes (*sox19b*, *sox2*, *sox3*) showed distinct maternal versus zygotic expression profiles across the six developmental stages (Supplementary Figure S1; Supplementary Table S5). *nanog* was highly abundant in unfertilized eggs and declined from blastula onwards, whereas *pou5f3* and *sox19b* were strongly expressed maternally and remained elevated through blastula and 25% epiboly before decreasing at 50 dd. *sox3* maintained stable high expression throughout development. In contrast, *sox2* showed low expression in unfertilized and cleavage-stage embryos and increased strongly from blastula to 50 dd, consistent with zygotic activation.

To place these dynamics in a broader developmental context, we examined maternal versus zygotic expression patterns for the 58 developmental patterning genes included in Table 1; Figure 10. Of these, 62% were classified as zygotically activated (low expression in unfertilized eggs with strong induction post-MBT), 14% showed both maternal provisioning and zygotic enhancement, and 21% were primarily maternally provided. Peak-expression timing showed a similar trend: 84% of genes reached maximal expression at or after the mid-blastula transition (BL, 25 EB, or 50dd), with nearly half (48%) peaking at 25% epiboly.

## 4 Discussion

Transcriptomic analysis of Atlantic halibut eggs is a valuable tool for identifying biological processes, single genes, and gene sets responsible for critical developmental events such as localization, body axis formation, and patterning of the developing embryo. This study provides the first comprehensive transcriptomic overview of early embryonic development in Atlantic halibut, addressing the previously unexplored temporal expression patterns of genes involved in these processes. These findings provide essential reference points for transcriptomic comparisons, such as in egg quality studies, by helping researchers select the developmental stages in which genes linked to poor phenotypes exhibit their highest activity.

Our analysis revealed distinct phases of transcriptional activity across early halibut development. Between UF and 16C, only limited transcriptional differences were observed, consistent with the dominance of maternal transcripts during early cleavage divisions (Lee et al., 2013; Vesterlund et al., 2011). Some developmental GO terms appeared higher in UF eggs, possibly reflecting ovarian fluid contamination or maternal transcripts associated with ongoing oogenesis, as reported in salmonids (Guzmán et al., 2014).

The most dramatic changes occurred during the 16C→BL transition, corresponding to the mid-blastula transition (MBT). At this stage, PCA revealed the largest spread among samples. This likely reflects both the rapid and heterogeneous transcriptional reprogramming that occurs during MBT and the practical difficulty of perfectly synchronizing sampling at this fast-changing stage, where small timing differences can translate into large transcriptomic differences. Canonical developmental pathways such as Wnt (GO:0016055), Notch (GO:0007219), and BMP signaling (GO:0030510) were strongly overrepresented, together with broad categories such as embryo development (GO:0009790) and embryonic morphogenesis (GO:0048598). This shift reflects the onset of zygotic genome activation (MZT) and is consistent with studies in zebrafish and other vertebrates showing sharp transcriptional activation at MBT (Kane and Kimmel, 1993; Vastenhouw et al., 2019).

From BL→25 EB, gastrulation was accompanied by overrepresentation of additional developmental processes such as pattern specification (GO:0007389), mesoderm development (GO:0007498), and left/right symmetry (GO:0007368), in line with morphogenetic reorganization of the embryo (Feldman et al., 1998). By the 25 EB→50 dd transition, transcriptional activity shifted toward later differentiation processes, including neuron differentiation (GO:0030182), muscle structure development (GO:0061061), and cranial skeletal development (GO:1904888), consistent with the onset of organogenesis after gastrulation in teleosts and lineage-specific maturation programs (Keenan and Currie, 2019). Collectively, these results show that while maternal transcripts support cleavage divisions, the decisive transcriptional engagement of axis and patterning programs is zygotically driven from MBT onwards.

The temporal profiles of zygotic genome activators and developmental patterning genes highlight the central role of the mid-blastula transition in Atlantic halibut development. Halibut orthologs of *nanog*, *pou5f3*, and several SoxB1 factors showed expression dynamics broadly consistent with zebrafish (Lee et al., 2013), with strong maternal provisioning and maintenance through early gastrulation, whereas *sox2* was predominantly zygotically activated, indicating functional divergence within the SoxB1 family. In zebrafish, large-scale surveys have classified transcripts into maternal, zygotic, and maternal-zygotic categories and tracked their decay or activation across the MZT (Baia Amaral et al., 2024; Harvey et al., 2013). Although our focus here is not to mechanistically assign transcript origin, but rather to determine when key developmental regulators become transcriptionally active in Atlantic halibut, the timing we observe, where most patterning genes rise sharply after the mid-blastula transition, is broadly consistent with these generalized dynamics. Across the 58 developmental patterning genes examined, 84 percent reached peak expression at or after the blastula stage, and 62 percent showed clear zygotic activation rather than maternal provisioning. This predominance of zygotic control helps explain why unfertilized-egg transcriptomes provide limited insight into developmental competence: most genes that pattern the embryo are not yet transcriptionally engaged, reinforcing that post-MBT stages represent the biologically informative window for assessing early developmental potential in Atlantic halibut.

Analysis of curated developmental genes further clarified the timing of axis specification. Maternal expression of *ctnnb1*, *hwa*, and *sybu* supported early dorsal organizer formation, consistent with Wnt/β-catenin–driven dorsalization in zebrafish (Kelly et al., 2000; Nojima et al., 2010; Yan et al., 2018). However, most DV and AP regulators, including *bmp2b*, *wnt8b*, *fgf8a*, *sp5a*, and *sp5l*, were transcriptionally silent before MBT and sharply upregulated at BL, highlighting that axis establishment is primarily a zygotic process (Challa and Chatti, 2013; Langdon and Mullins, 2011; Weidinger et al., 2005). A few later-expressed genes (*spaw, dand5*) marked the onset of left–right asymmetry at 50 dd (Hashimoto et al., 2004; Long et al., 2003). Taken together, these dynamics indicate a conserved division of labor where a handful of maternal Wnt regulators initiate dorsal identity, but zygotic activation of Wnt, BMP, FGF, and Notch cascades drives the establishment of DV and AP axes and organizes gastrulation movements. This echoes findings in zebrafish and other teleosts, underscoring the evolutionary conservation of MZT as the true onset of developmental patterning (Dorey and Amaya, 2010; Fürthauer et al., 2004).

Many aquaculture studies of egg quality have relied on transcriptomic or proteomic comparisons of unfertilized or very early cleavage-stage eggs (Bonnet et al., 2007; Mommens et al., 2010; Mommens et al., 2014; Yilmaz et al., 2022; Żarski et al., 2017). While these approaches have identified differences in maternal transcript or protein abundance, our data show that the core developmental programs governing axis formation and morphogenesis are not yet active at these stages. Instead, the decisive transcriptional activity begins at MBT and gastrulation. This conclusion aligns with findings in channel catfish, where Myers et al. (2020) tracked gene expression across multiple developmental stages and reported that differences between good- and poor-quality eggs were minimal in unfertilized and cleavage stages but became pronounced at neurulation (48 hpf). These results and the present study highlight that transcriptomic signatures of egg quality may only emerge at specific developmental transitions, rather than at the earliest stages.

While large-scale transcriptional activation in Atlantic halibut begins during the MBT, maternal transcripts likely exert important preparatory roles that influence how zygotic programs unfold. Maternal factors are known to shape early chromatin landscapes and establish transcriptional competence prior to zygotic genome activation, for example, through chromatin remodeling and pioneer factor activity (Gentsch et al., 2019; Hontelez et al., 2015). In other vertebrates, maternally deposited enzymes such as histone demethylases and other epigenetic regulators also contribute to proper reprogramming of the embryonic genome (Ancelin et al., 2016). Such maternal provisioning may therefore pattern the molecular environment that enables robust zygotic activation. From an applied perspective, this suggests that while later developmental stages offer clearer transcriptomic signatures for assessing egg or embryo quality, variation in maternal inputs could still influence these outcomes indirectly through early epigenetic or regulatory priming.

## 5 Conclusion

This study provides a stage-resolved transcriptomic overview of early Atlantic halibut development, from unfertilized eggs through blastopore closure. We identified critical temporal expression patterns and stage-specific activation of canonical developmental pathways, including Wnt, BMP, and FGF signaling, which underlie axis formation, germ layer specification, and morphogenetic reorganization. Maternal transcripts dominated cleavage stages and contributed to dorsal organizer establishment, but the decisive activation of body axis and patterning programs occurred after the mid-blastula transition. These findings underscore that transcriptomic differences relevant to egg quality may not be detectable in unfertilized or very early cleavage stages but instead emerge during and after MBT, when the embryo assumes transcriptional control. By resolving when key developmental programs are active, this dataset provides a reference framework for future egg quality studies and highlights candidate windows where transcriptomic biomarkers of embryonic competence and deformity risk could be most informative. While our study provides comprehensive temporal resolution of transcript abundance, spatial localization data through techniques such as RNA *in situ* hybridization would further validate regional expression of axis patterning genes. Future studies combining this transcriptomic baseline with spatial techniques would provide a complete picture of halibut embryonic patterning.

## Data Availability

The data presented in the study are deposited in the BioStudies repository, accession number E-MTAB-15858.
