# Supplementary figures and images for: Maternal provisioning and zygotic activation: transcriptomic dynamics of early atlantic halibut (Hippoglossus hippoglossus) embryogenesis
Source: Front Cell Dev Biol. 2025 Dec 8;13:1723170. doi: 10.3389/fcell.2025.1723170 (PMC12722812; doi:10.3389/fcell.2025.1723170)

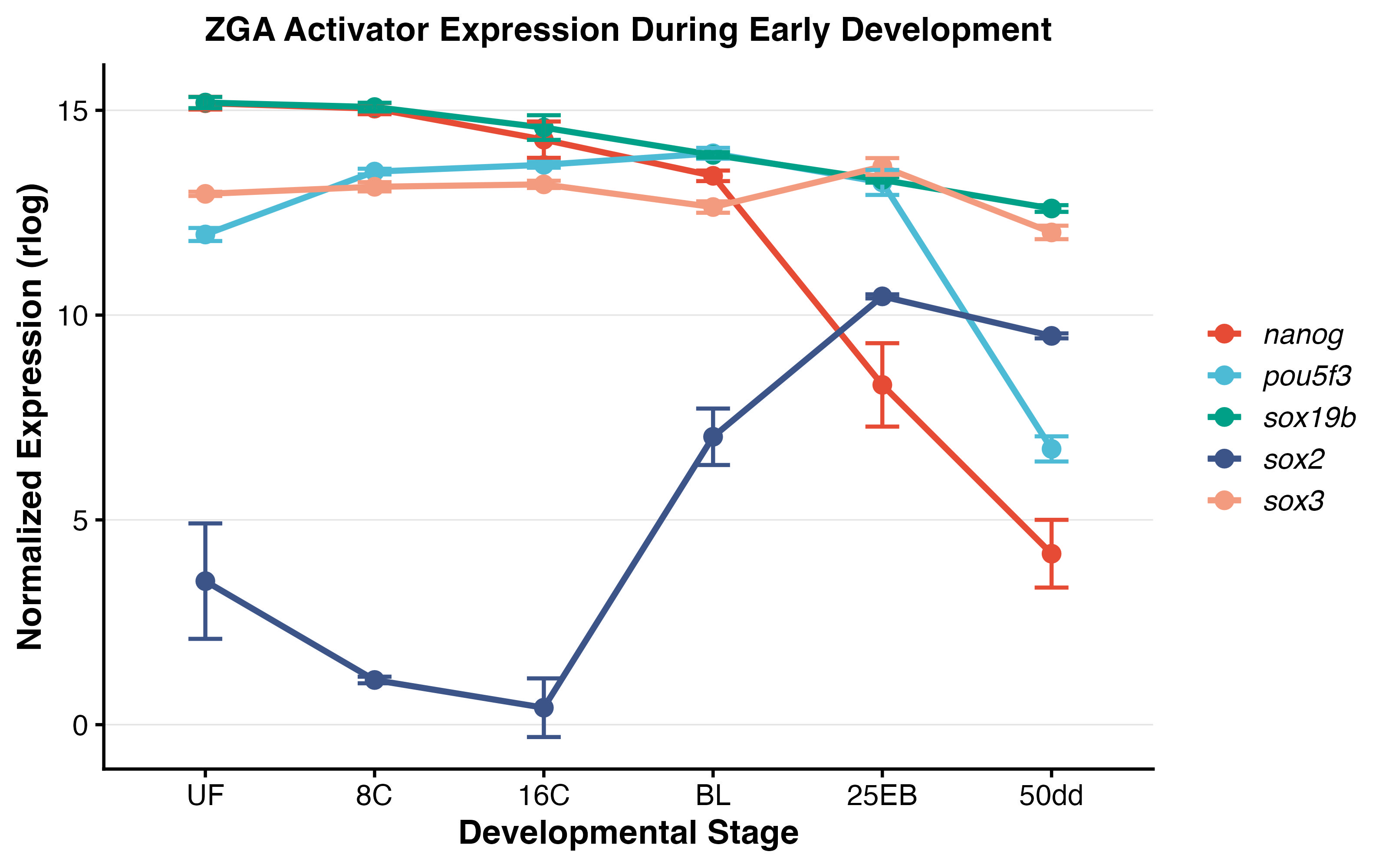

Supplement: Supplementary file 3 [file Image1.jpeg]
